# The acceptance and applicability of a patient-reported experience measurement tool in oncological care: a descriptive feasibility study in northern Germany

**DOI:** 10.1186/s12913-019-4646-4

**Published:** 2019-11-01

**Authors:** Christiane Rudolph, Gitte Stentebjerg Petersen, Ron Pritzkuleit, Hans Storm, Alexander Katalinic

**Affiliations:** 1 Institute for Cancer Epidemiology, University of Lübeck, Ratzeburger Allee 160, 23562 Lübeck, Germany; 2 Danish Cancer Society, Strandboulevarden 49, 2100 København, Denmark

**Keywords:** Breast cancer, Colorectal cancer, Patient-centered care, Patient preference, Patient satisfaction, Quality of health care, Pilot study

## Abstract

**Background:**

Patient-reported outcome measures (PROMs) and patient-reported experience measures (PREMs) are tools for assessing outcomes of and experiences with health care from the patient’s perspective. In Germany, PROMs are widely used in research for evaluating patient outcomes and quality of care. However, the application of PREMs is rather scant, especially in oncology. The study aimed to assess the feasibility of patient-centred quality evaluation in oncological care in Germany using the German adaptation of the Danish National Cancer Patient Questionnaire. This questionnaire is a PREM/PROM-tool addressing patients of all cancer sites and covering the entire cancer patient pathway.

**Methods:**

The Danish National Cancer Patient Questionnaire was translated into German via forward-backward translation. Face-validity was tested among three cancer patients in a conventional pre-test. The German adaptation contains 99 questions. A pilot test was carried out among 245 newly diagnosed breast and colorectal cancer patients in the German federal state Schleswig-Holstein. Patients were recruited via clinics participating in the Oncological Care Registry (12 specialised units in seven hospitals) and contacted six to nine months after diagnosis. Response behaviour and response patterns were compared to the Danish study population (*n* = 1964).

**Results:**

The willingness among clinicians to support patient recruitment as well as the response rate of patients to the questionnaire was high (65%). Moreover, response behaviour and response patterns of German and Danish patients were consistent. Despite the generally good response behaviour of patients to the single items, the authors observed that questions assessing the diagnostic process did not fully capture German pathways. Only 19.3% of the German patients stated that their diagnostic process was initiated by a visit to a general practitioner (GP) in contrast to 52% in Denmark. The assessment of patient experiences in the diagnostic phase heavily focuses on experiences in general practice, which does not seem appropriate in the German health care setting.

**Conclusion:**

The translation was successful, and the feasibility of a future large-scale study within existing structures is given. However, some modifications of questions heavily related to the Danish health care system, especially referring to the diagnostic phase, are necessary.

## Background

The patient’s perceptive is of paramount importance for the assessment of health care quality and essential for providing patient-centred care. Patient evaluations are the only source of information about health care quality with regard to several aspects of care such as information provided, communication and interaction with medical staff or quality of life. Patient-reported measures have thus gained increasing importance in the evaluation of health care in addition to, for example, clinical quality indicators [1, 2]. There are two categories of patient-reported measures: Patient-reported outcome measures (PROMs) and patient-reported experience measures (PREMs). While PROMs measure health care outcomes (e.g. side effects or health-related quality of life), PREMs assess patient’s needs and experiences whilst receiving care (e.g. involvement in care decision making or accessibility of services) [3].

In Germany, PROMs, especially quality of life measures, are widely and routinely used across various medical disciplines [4–8] including oncology [9–13]. The application of PREMs, however, is rather scant, especially in oncological care. Most studies focus on patient experiences in either outpatient [14, 15] or inpatient [16–20] care settings using generic instruments. Patient surveys designed specifically to assess cancer patients’ experiences are scarce in Germany and limited to inpatient care of a specific cancer site [21] or to outpatient care [22]. Most instruments are limited to hospital care, which is not sufficient in cancer care. The diagnostic phase is often initiated in general practice or specialised outpatient care [23], and also rehabilitation is a significant part of the cancer trajectory [24, 25]. In fact, a systematic review showed that cancer patients rate the diagnostic phase as the most important dimension of cancer care [26]. Thus these aspects have to be included in the evaluation of cancer care quality. To our knowledge, there is no publicly available PREM-tool in the German language that addresses cancer patients of all cancer sites and covers the entire cancer patient pathway from initial contact to a health care service provider until discharge from hospital. Such a tool would allow identification of care aspects along the entire cancer trajectory, where patient preferences and needs are inadequately addressed. As patient-centred health care and positive patient experiences are associated with better adherence to treatment and thus with better outcomes, the utilisation of such a survey instrument and the reporting of patient experience data is an essential step towards improving cancer care [27, 28].

Since 2009, The Danish Cancer Society have developed and adjusted/improved tools for assessing patient-reported experiences and outcomes in oncological care throughout the entire patient pathway [29, 30]. In 2017, a survey based on the Danish National Cancer Patient Questionnaire was conducted among newly diagnosed cancer patients. The aim was to explore cancer patients’ needs and experiences regarding diagnostic care and treatment in Denmark [31]. The same year the Danish questionnaire was translated into German. A pilot study using the German adaptation was carried out among newly diagnosed patients with breast cancer and colorectal cancer in the northernmost German federal state Schleswig-Holstein. The aim of this study was i) to assess the feasibility of patient-centred quality evaluation in oncological care in Germany within existing structures ii) to explore the acceptance and applicability of the German adaptation of the Danish National Cancer Patient Questionnaire in the German health care setting and iii) to collect the initial information on patient-reported experiences in Schleswig-Holstein in comparison to Denmark.

## Methods

In the German federal state Schleswig-Holstein, a feasibility study was conducted using the German adaptation of the Danish National Cancer Patient Questionnaire (2017-version).

### The Danish cancer patient questionnaire

The Danish National Cancer Patient Questionnaire (2017-version) is a self-administered data collection tool containing 129 questions of which several are multi-item scales. The questionnaire covers the entire cancer patient pathway from diagnosis over treatment to discharge from hospital. The tool was developed by the Danish Cancer Society in 2010 [29]. The first version of the tool was extensively revised in 2016–2017 in a multi-stage process [31], including:

- Literature search in PubMed and Google was carried out in order to identify relevant topics for patient-centred health care evaluation among cancer patients in addition to relevant topics included in previous versions of the tool.
- An online survey (533 cancer patients responded), focus groups and individual interviews were conducted to identify important issues or aspects encountered in cancer care to strengthen the patients’ perspective in the questionnaire development.
- Patient representatives, clinicians and researchers summarised the identified topics from literature search, patient survey and interviews and critically evaluated the items to be included in the questionnaire.
- A pilot test was conducted among 559 cancer patients to validate 18 new PREMs included in the 2017-version. Furthermore, 22 cognitive interviews were conducted among cancer patients in order to validate the whole questionnaire.

### Translation and adaptation of the Danish tool

Two-thirds of the Danish questionnaire consist of PREMs and one-third includes a range of validated PROM-scales such as the EORTC-QLQ-C30 Quality of Life assessment tool and sub-scales of the Work Ability Index (WAI) and Health Literacy Questionnaire (HLQ), for which validated German translations are already available [32–34]. We identified questions that were specifically targeting the Danish health care system or Danish research collaborations and thus were not applicable to Germany. Additionally, nineteen questions (all PREMs) were removed from the questionnaire in order to slightly shorten the extensive tool. For the remaining questions, that were to be included in the German version and for which no German translation was already available, a forward-backward translation was conducted in order to obtain a translation as valid as possible. The procedure of a forward-backward translation involved a translation from Danish to German by a project member (CR) and a back-translation from German to Danish by a third independent person (professional translator). Thereafter an expert panel including researchers and a communication consultant working in the project compared the two Danish versions and derived at a more precise German translation of the questionnaire. Some questions and their respective response categories had to be adapted to the German health care and social security system. Subsequently, a conventional pre-test among three cancer patients was conducted, which did not lead to major changes in the translation and established face validity. The German version contains 99 questions of which several are multi-item scales resulting in a total of 157 items on 20 pages. It takes approximately an hour to fill out the questionnaire (Additional file 1: Figure S1).

### Feasibility outcomes of the pilot study in Schleswig-Holstein, Germany

Feasibility outcomes were defined and operationalised in accordance with the study objectives as follows: i) The feasibility of a patient-centred quality assessment in oncological care within the existing structures of the health care system was assessed on the basis of the willingness of the contacted hospital departments to support the study by recruiting patients. ii) Acceptance and applicability of the German adaptation of the Danish National Cancer Patient Questionnaire in the German health care setting were assessed by response rate, proportion of missing values and comparison of response patterns across countries. iii) Additionally, initial descriptive comparisons of patient-reported experiences in Schleswig-Holstein and in Denmark elicited information on the consistency of responses.

### Data collection in Germany and Denmark

The German adaptation of the questionnaire was tested among colorectal and breast cancer patients in Schleswig-Holstein, Germany. The patients were 30 to 99 years old and newly diagnosed between January and March 2017. Patients with carcinomas in situ and male breast cancer patients were not included in the study. The patients were recruited via clinics in the Oncological Care Registry (Onkologisches Versorgungsregister) in Schleswig-Holstein. We invited eight hospitals with thirteen specialised units to participate in the pilot study. Within existing patient data exchange routines in the oncological care registry, patient contact information as well as information on sex, age, date of diagnosis and cancer site (diagnosis according to the ICD-10) were delivered by the hospitals for all breast and colorectal cancer patients treated at the respective hospital fulfilling the above mentioned in- and exclusion criteria. The questionnaire was sent out by the cancer registry to 245 patients six to nine months after diagnosis. In contrast to the Danish survey, patients could fill out and send back a paper-based version of the questionnaire only. After three weeks, patients who had not responded by then were invited once again to participate. Ethical approval for the German pilot study was obtained from the Ethical Committee at the University of Lübeck.

Data collected in the German pilot study was compared to results of the Danish nationwide population-based survey, which was conducted among cancer patients aged 30 to 99 years. Patients were identified through the National Patient Registry [35] and included patients registered with a cancer diagnosis for the first time between July and December 2016. The Danish National Cancer Patient Questionnaire was distributed four to seven months after diagnosis. Patients could either fill out and send back the paper-and-pencil version or use the web-based survey. For comparability with the German survey, we restricted the sample from Denmark to female breast cancer cases and colorectal cancer cases of both sexes. In total, 3399 Danish breast and colorectal cancer patients were eligible and received a questionnaire. The Danish study was approved by the Danish Data Protection Agency. Under Danish law, questionnaire studies are not subject to review by the Ethics Committee System.

Within the scope of this article, we focus on patient-reported experience measures and exclude further exploration of validated scales for the assessment of patient-reported experiences and outcomes (HLQ, WAI, EORTC-QLQ-C30) in the Results and Discussion section.

### Statistical analysis

We present overall response rates, response rates by cancer site and proportions of missing values in the German and the Danish sample for key questions. Moreover, frequency tables of patient reports and ratings of cancer care are shown for selected key questions. Descriptive statistics were performed in SAS 9.4 (Danish analysis) and IBM SPSS Statistics Version 22 (German analysis). No inferential statistics were performed as no hypothesis was tested. All results are exploratory.

## Results

### Feasibility, acceptance and applicability

All contacted hospitals but one were willing to support the study in terms of patient recruitment (12 specialised units in seven hospitals out of 13 specialised units in eight hospitals participated). One hospital declined due to the additional administrative burden.

In total, 65.3% (*n* = 160 out of 245) of the German patients responded to the postal questionnaire (Table 1). The response rate was slightly higher than in the Danish survey (response rate = 57.8%; *n* = 1964). In both samples, response rates among breast cancer patients are higher than among colorectal cancer patients. More patients in the German sample reported to have completed treatment and to suffer from comorbid conditions than in the Danish survey.

**Table 1:** Basic characteristics of the study participants in Schleswig-Holstein (SH), Germany and Denmark (DK)

	Schleswig-Holstein (SH), Germany			Denmark (DK)		
	Total		Response	Total		Response
	n	%	%	n	%	%
Total	160	100.0	65.3	1964	100.0	57.8
Cancer site						
Breast	115	71.9	68.5	1095	55.7	59.5
Colorectal	45	28.1	58.4	869	44.3	55.7
Sex^*^						
Female	21	46.6	60.0	359	41.3	52.7
Male	24	53.3	57.1	510	58.7	58.1
Age distribution of breast cancer patients						
30–49	16	13.9	61.5	168	15.3	51.2
50–59	34	29.6	82.9	245	22.4	60.2
60–69	31	27.0	67.4	332	30.3	63.6
70–79	28	24.3	70.0	246	22.5	65.6
80–99	6	5.2	40.0	104	9.5	50.0
Age distribution of colorectal cancer patients						
30–59	11	24.4	73.3	115	13.2	45.6
60–99	34	75.5	54.8	754	86.8	57.7
Self-reported treatment status						
Completed	109	68.1	–	1027	52.3	–
Ongoing	42	26.3	–	805	41.0	–
Unknown	9	5.6	–	132	6.7	–
Self-reported comorbid conditions						
Yes	85	53.1	–	813	41.2	–
No	57	35.6	–	950	48.4	–
Unknown	18	11.3	–	201	10.2	–

^*^colorectal cancer cases only

The missing values analysis showed that the proportion of missing answers to questions ranged from 0.0 to 45.1% with an average of 7.0% missing values per question (for detailed information on key questions please see Table 2). Those values are comparable to the results from the Danish survey (data not shown). The highest non-response rate of 45.1% was observed in a sub-question asking whether complications assessed in the previous question (Have you ever experienced any of the following problems: problems with chemotherapy (e.g. wrong doses or the chemo went outside the vein), not enough treatment against pain, occurrence of inflammation/infection or bedsores/pressure sores, etc.) had any consequences for the patient such as insecurities or prolonged hospitalisation.

**Table 2:** Proportion of missing values (MV) for key questions from the questionnaire

	% of MV	
	SH	DK
Diagnostic delay and satisfaction with diagnostic phase		
1. How did the process start that led to your diagnosis with cancer?	12.5	1.5
2. How do you rate the length of time:		
a) From your first examination by the general practitioner, until you were referred to a specialised doctor or hospital?	18.5	10.8
b) From your referral to examination at a specialised doctor or hospital, until you were diagnosed with cancer?	16.9	10.6
c) From when you were told that you had cancer until you received your first treatment?	1.3	3.5
3. Do you feel you were taken seriously when you attended the general practitioner with your symptoms?	7.7	5.3
4. Overall, how do you rate your diagnostic phase (i.e. what was done until you received your diagnosis)?	6.9	6.2
5. Do you feel your cancer diagnosis was given to you in a proper manner?	1.3	1.2
6. Is there anything you would have liked to have been different in the period of time from when you were told that you had cancer until your treatment was initiated?	1.9	3.0
Information & involvement		
7. Before you started treatment, did a doctor or nurse give you the information you needed in relation to:		
a) Your disease?	3.8	2.8
b) Your treatment(s)?	11.3	8.8
c) Which complications may occur after operation? (e.g. inflammation, bleeding)	6.9	9.8
d) Which side effect(s) of medicine may occur? (e.g. nausea, sensory disturbance after chemotherapy)	7.5	9.2
8. Were you sufficiently involved in treatment decisions? *Assessed* via *agreement or disagreement of answers to the following questions:*	20.0	15.0
a) Which statement describes best, how decisions about your treatment were made?*	11.3	7.6
b) Which statement describes best, how you would prefer decisions about your treatment are made?*	16.9	13.0
9. Have you ever doubted whether the treatment you were offered was the right treatment for you?	3.8	4.4
10. Do you feel that the doctors		
a) Treated you like a whole person and was not just interested in your disease?	5.7	6.6
b) Took time to understand what was important for you?	6.3	10.5
Continuity of care		
11. Which of the following statements best describes your experience at the hospital? *One particular doctor was responsible for my overall treatment,* etc. (see Additional file 2: Table S1)	7.6	8.6
12. Did you feel that there was a clear plan for your overall treatment pathway?	1.9	4.3
13. How do you rate the number of doctors you have been in contact with during your treatment at hospital?	1.9	4.4
14. Have you had at least one doctor at the hospital you felt that you could reach out to if you needed it?	1.3	6.6
Help & support		
15. Have you received the help you needed from the healthcare system/municipality in relation to:		
a) Physical rehabilitation?	8.8	8.4
b) Anxiety, sadness or worries (e.g. talks with i.e. a psychologist/priest)?	10.0	10.2
c) Unexpected weight changes/malnutrition (e.g. dietary regimen, dietary advice)?	9.4	10.9
d) Problems with intimacy or relationships?	11.9	11.0
Discharge and overall treatment		
16. Did you feel comfortable being discharged from hospital?	0.9	6.7
17. Did you get the information you needed about which symptoms are important for you to respond to?	1.8	9.4
18. Do you know who you can contact at the hospital if you need to?	0.9	6.2
19. Overall, how do you rate your overall care and treatment at hospital?	2.5	5.0

*Patients could choose between following statements:

A. I make the decisions about which treatment I will receive

B. I make the decisions about which treatment I will receive after considering my doctor’s opinion

C. The doctor and I make the decisions together about which treatment I will receive

D. The doctor makes the decision about which treatment I will receive, but he/she seriously considers my opinion

E. The doctor makes the decision about which treatment I will receive without involving me

We observed that questions asking about the diagnostic process did not fully capture German pathways. Less than a fifth of the German patients stated that their diagnostic process was initiated by a visit to a general practitioner (GP) in contrast to 52% in Denmark (Table 3). When patients were asked about the timely appropriateness of referrals from GP to specialised care, high missing values were observed in the German study population (Table 2). In general, the assessment of patient experiences in the diagnostic phase heavily focuses on experiences in general practice. Though there are questions filtering for being in contact with the GP in order to avoid that patients answer to questions that are not relevant for them, these were partly ignored by patients or questions about GPs were answered because patients contacted them for other reasons than the suspected cancer disease. Despite these difficulties, respondents showed generally good response behaviour.

**Table 3:** Patient-reported experiences during the diagnostic phase and satisfaction with the diagnostic phase

Diagnostic delay and satisfaction with diagnostic phase	SH	DK
	%	%
1. How did the process start that led to your diagnosis with cancer?	*n* = 140	*n* = 1935
- I was in contact with my own general practitioner (GP)	19.3	52.3
- I was in contact with a specialist outside of the hospital (e.g. gynaecologist)	26.4	1.1
- I attended cancer screening (e.g. breast-, cervix- or colorectal cancer)	30.7	33.3
- I was treated for another disease at the hospital	5.0	2.9
- I was hospitalized as the result of an emergency	2.1	3.2
- Other	16.4	7.2
2. How do you rate the length of time from:		
a) examination by the GP until referral to a specialised doctor or hospital?	*n* = 53	*n* = 973
- Adequate	84.9	74.8
- Too long	9.4	12.2
- Too short	0.0	9.9
- Not relevant	5.7	3.0
b) referral to examination at a specialised doctor/hospital until receiving the diagnosis?	*n* = 54	*n* = 975
- Adequate	75.9	72.2
- Too long	11.1	15.2
- Too short	1.9	9.9
- Not relevant	11.1	2.8
c) cancer diagnosis until you received your first treatment?	*n* = 158	*n* = 1896
- Adequate	86.1	79.4
- Too long	11.4	13.8
- Too short	1.9	3.9
- Not relevant	0.6	3.0
3. Do you feel you were taken seriously when you attended the GP with your symptoms?	*n* = 60	*n* = 1033
- Yes, to a great extent	63.3	76.4
- Yes, to some extent	10.0	10.8
- To a lesser extent	8.3	4.5
- No, not at all	5.0	6.2
- Not relevant	13.3	2.2
4. Overall, how do you rate your diagnostic phase?	*n* = 149	*n* = 1843
- Particularly good	40.3	56.5
- Good in the main	51.7	33.2
- Poor in the main	4.0	4.1
- Particularly poor	2.7	2.2
- Not relevant	1.3	4.1
5. Do you feel your cancer diagnosis was given to you in a proper manner?	*n* = 158	*n* = 1941
- Yes, to a great extent	62.7	74.2
- Yes, to some extent	25.3	19.2
- To a lesser extent	8.2	4.0
- No, not at all	3.8	2.7
6. Is there anything you would have liked to be different?	*n* = 157	*n* = 1904
- No, there was nothing else I would have liked	71.3	73.5
- Yes	28.7	26.5
Among those, who stated yes, would have liked		
- to have started treatment sooner (e.g. operation, chemotherapy)	53.3	68.5
- to have waited a little longer before I started treatment	2.2	2.4
- more information before I started treatment	28.9	15.1
- a more in depth conversation before I started treatment	26.7	15.5

### Patient experiences and needs

In total, we analysed 99 questions/multiple item scales with overall 157 items. Tables 3, 4 and 5 and Additional file 2: Table S1 and Additional file 3: Table S2 show the selected questions and answers for the German sample and the Danish sample. Questions were partly abbreviated for better readability of the tables. For original wording please check Table 2.

**Table 4:** Patient-reported experiences on information need and involvement in care decision making

Information & involvement in decision making	SH	DK
	%	%
7. Did you get the information you needed in relation to:		
a) Your cancer disease?	*n* = 154	*n* = 1909
- Yes, had enough information	87.0	89.3
- Lacked a little information	9.1	7.2
- Lacked a lot of information	2.6	1.2
- Had too much information	0.6	0.6
- Not relevant	0.6	1.7
b) Your treatment option(s)?	*n* = 142	*n* = 1791
- Yes, had enough information	83.1	86.2
- Lacked a little information	12.7	8.9
- Lacked a lot of information	3.5	1.8
- Had too much information	0.0	0.4
- Not relevant	0.7	2.7
c) Which complications may occur after operation?	*n* = 149	*n* = 1772
- Yes, had enough information	81.2	75.2
- Lacked a little information	10.7	12.0
- Lacked a lot of information	4.0	4.9
- Had too much information	0.7	0.1
- Not relevant	3.4	7.8
d) Which side effects of medicine may occur?	*n* = 148	*n* = 1784
- Yes, had enough information	69.4	70.3
- Lacked a little information	14.2	9.4
- Lacked a lot of information	6.1	4.3
- Had too much information	0.0	0.8
- Not relevant	10.1	15.2
8. Were you sufficiently involved in treatment decisions?^*^	*n* = 128	*n* = 1670
- Sufficiently involved	79.7	80.1
- Involved too much	3.1	6.3
- Involved too little	17.2	13.6
9. Have you ever doubted whether your treatment was the right treatment for you?	*n* = 152	*n* = 1865
- No, not at all	62.5	75.0
- To a lesser extent	8.6	9.0
- Yes, to some extent	15.8	8.5
- Yes, to a great extent	12.5	6.7
- Not relevant	0.7	0.9
10. Do you feel that the doctors:		
a) Treated you like a whole person and were not just interested in your disease?	*n* = 149	*n* = 1821
- Yes, all of them	52.3	64.5
- Yes, most of them	36.2	23.6
- Some of them	8.1	8.4
- No, none of them	1.3	1.4
- Don’t know	2.0	2.1
b) Took time to understand what was important for you?	*n* = 148	*n* = 1746
- Yes, all of them	50.0	55.8
- Yes, most of them	34.5	26.6
- Some of them	11.5	11.1
- No, none of them	3.4	2.2
- Don’t know	0.7	4.3

*The results are based on a calculation of agreement or disagreement between patients’ preferences of involvement and patients’ experiences of involvement (for original questions see Table 2)

**Table 5:** Patient-reported need for support and degree to which the need was met

Did you receive the help you needed from health services in relation to:		Had a need	Met need among those who had a need			
			Yes, to a great extent	Yes, to some extent	To lesser extent	No, not at all
Physical rehabilitation	SH (*n* = 146)	57.5%	33.3%	34.5%	17.9%	14.3%
	DK (*n* = 1799)	45.4%	51.8%	23.2%	7.8%	17.2%
Anxiety, sadness or worries	SH (*n* = 144)	36.8%	30.2%	26.4%	22.6%	20.8%
	DK (*n* = 1763)	30.4%	15.5%	15.1%	16.5%	52.9%
Unexpected weight changes/malnutrition	SH (*n* = 145)	34.5%	18.0%	18.0%	28.0%	36.0%
	DK (*n* = 1750)	34.3%	16.3%	18.5%	18.8%	46.3%
Problems with intimacy or relationships	SH (*n* = 141)	19.9%	10.7%	7.1%	7.1%	75.0%
	DK (*n* = 1747)	24.6%	5.8%	14.4%	20.7%	59.1%

The majority of cancer patients rated the time from examination at the GP until referral to specialised care as adequate. Roughly 10% of the survey participants stated that it took too long. Interestingly, 10% of the Danish breast and colorectal cancer patients stated that the time in-between examination and referral was too short. Similar patterns were observed regarding the timeliness of diagnosis after examination at specialised care and for initial treatment after diagnosis. More Danish patients felt taken seriously when they presented their symptoms at the GP than German patients, though considerably more German patients stated that this aspect was not relevant to them. The majority of patients were satisfied with the way the cancer diagnosis was delivered. More than 70% of the participants stated that there was nothing they would have liked to have been different in the period from when they were diagnosed with cancer until treatment was initiated. Of those who indicated potential for improvement, most would have liked to have started treatment sooner (Table 3).

The information need was largely met, though a slight tendency towards the need for more information was observed especially regarding treatment side effects. In both samples, roughly 80% of the patients indicated that they were sufficiently involved in care decision making. In Denmark, 6.3% of the study participants stated that they were involved too much, while 13.6% reported to have been involved to a lesser extent than they had preferred. A similar trend was observed in Germany (3.1 and 17.2%). The proportion of patients expressing doubt about the appropriateness of offered treatment was also slightly higher among the German cancer patients. The majority of respondents reported that the doctors treated them like a whole person, and were not just interested in the disease and took the time to understand what was important to the patients. Contentment, however, was higher among Danish patients (Table 4).

One fifth of the German respondents and almost a third of the Danish patients reported that they experienced uncertainty with respect to the responsibility of doctors and they stated that they were not always informed about which doctor was responsible for their care or they felt no doctor at all was responsible. Almost 30% of the Danish breast and colorectal cancer patients stated that (slightly) too many doctors were involved, whereas only 12% of the German patients reported this (Additional file 2: Table S1).

The need for support was highest in relation to physical rehabilitation followed by dealing with anxiety, sadness or worries and handling unexpected weight changes/malnutrition. The need for physical rehabilitation was largely met, though German cancer patients reported slightly more often than Danish patients that their need was not met adequately. With regard to support in coping with anxiety, sadness or worries, 44% of the German respondents, who indicated a need, stated that their need was not met. A by far larger proportion of 70% of the Danish respondents indicated that their need of support in coping with mental distress was not met. The need for support in relation to unexpected weight changes/ malnutrition and problems with intimacy or relationships were largely unmet in both samples (Table 5).

Though the vast majority of patients felt comfortable to be discharged from hospital, more than 20% of the participants reported, that they did not receive enough information regarding symptoms that need to be reacted to. A similarly high proportion of patients stated, that they do not know, whom they could contact if necessary. Overall however, more than 95% of the patients rated their care at hospital as particularly good or good in the main (Additional file 3: Table S2).

## Discussion

To our knowledge, this is the first study assessing the feasibility of collecting data with patient-reported experience measures covering the entire patient pathway from diagnosis to discharge from hospital and eliciting information on patients’ needs and preferences in oncological care in Germany.

We observed high interest among leading hospital staff to assess patient-reported experiences and outcomes. This resulted in a high participation rate of hospital departments contacted for study purposes. Moreover, the high response rate (65.3%) in this survey despite the use of an extensive questionnaire is indicative of relevance for and good acceptance among cancer patients. In contrast to our results, studies report lower response rates to postal surveys on an average of approximately 50–60% in organisational [36] and medical research [37] with a trend towards declining survey response rates in more recent years [38]. Factors positively influencing response rates are among others high relevance of the surveyed topic to the study population and the implementation of reminder letters [39, 40] as done in this study.

Many similarities regarding (non-)response patterns and self-reports from German and Danish patients were observed, but where differences were noted they appear to be plausible. The outlier non-response rate of 45.1% to a sub-question assessing consequences of complications, which were assessed in the question before, was probably caused by questionnaire layout separating the two questions with a page break. Patients may not have understood what the sub-question was referring to and thus skipped answering it. In future studies such inapt layout will be avoided. Differences were observed regarding treatment status of patients with more German than Danish patients reporting to have completed treatment. As the survey was conducted slightly later in Schleswig-Holstein (6–9 months after diagnosis) than in Denmark (4–7 months after diagnosis), this finding was expected. When asked about the timely appropriateness of referrals from GP to specialised care, high proportions of missing values were observed among the German cancer patients. This might be explained by the fact that referrals from GPs to specialised doctors are wanted but not mandatory in the German health care system and patients can directly consult a specialist whereas GPs have a gatekeeper function in Denmark for referrals to specialised care. Likewise, a rather large proportion of German patients stated that the aspect of having felt taken seriously when they presented their symptoms at the GP was not relevant to them. It is possible that respondents skipped the filter question regarding initial contact to the GP during the diagnostic phase and answered the following GP-related questions despite not having been in contact with their GP at all or they had been in contact with their GP but for other reasons than their suspected cancer. Overall, only 19.3% of the German respondents replied that the process leading to their cancer diagnosis was started by a visit to the GP. Thus it seems not to be appropriate to focus on the patient-GP-relationship during the diagnostic phase in Germany. A more thorough pre-test e.g. including cognitive interviews, might have elicited this aspect, which the conventional pre-test failed to identify. However, this observation is limited to breast and colorectal cancer patients and the proportion of patients consulting a GP might be higher among patients of other cancer sites for which no screening programmes are available. In future work, we will shorten but not eliminate the extensive GP-related section of the questionnaire. Another remarkable finding is that unlike German patients, 10% of the Danish cancer patients stated that the time in-between examination at the GP and referral as well as in-between examination at a specialist and diagnosis were too short. More in-depth analyses exploring qualitative statements from Danish patients showed that many patients did not find the category “adequate” positive enough, and therefore answered “too short”. Thus there are reasons to doubt the validity of responses of Danish patients to these items. Another major difference observed was a considerably higher proportion of Danish patients reporting to have met too many doctors and to have experienced no clear doctor responsibility in comparison to German patients. Higher contentment with regard to the continuity of care among German patients might be explained by the unique structure of German hospitals included in this study. In Schleswig-Holstein, it is common to pool cancer care for the most common cancers at cancer site-specific clinics, so called “cancer centres”, which offer more comprehensive care (e.g. multidisciplinary tumour board reviews) than usual hospitals. This way of organising cancer care might result in smoother continuity of care. Likewise, a much lower proportion of German cancer patients reported unmet needs of support in coping with anxiety and depression than Danish patients. This might be a result of both the more specialised care at cancer centres and the potentially higher general awareness of the psycho-oncological burden for cancer patients among German physicians as there are clinical guidelines for psycho-oncological care available in Germany [41] but unfortunately not yet in Denmark.

Overall, the cancer patients evaluated oncological care positively across countries. This finding is not surprising as patient satisfaction and experience surveys often are criticized for producing skewed positive results [42, 43], which reduce opportunities to support changes or improve health care. This survey however, presents more useful results due to the development of a tool with questions specifically targeting cancer patients in contrast to generic tools applied to cancer patients. Rather than asking for general patient satisfaction this survey collects information about actual experiences or specific events e.g. lack of information about treatment options, level of involvement in decisions, insufficient pain management and unmet psychosocial needs and thus enabling targeted quality improvement.

The analysis of this survey, however, focused on descriptive statistics and no analytical statistical tests were performed as the comparisons between the non-representative German sample and the representative Danish sample do not allow generalisable conclusions. Also stratified analyses, e.g. cancer site specific analysis, were not conducted due to the small German sample. Furthermore, the proportion of breast cancer patients was significantly overrepresented in the German population with 72% of the sample being women with breast cancer compared to 56% in the Danish sample. Thus, observed differences in evaluated quality of care across countries could partly be due to persistent differences in the quality of care as experienced by breast cancer patients and colorectal cancer patients with breast cancer patients tending to rate care more positively as shown in previous work [44]. Nonetheless, the non-representative German sample might still be indicative of trends and shed light on aspects that might be worth investigating further. As the cancer patients enrolled in this survey were treated at cancer centres, the sample might not be entirely representative of all cancer patients with breast and colorectal cancer since other forms of health care provided in specialised outpatient care or university-level medical care are not included. It needs to be investigated whether the higher degree of needs met in psycho-oncological care in Germany compared to Denmark can be attributed to surveying patients treated at hospitals with more comprehensive cancer care or if these cross-border differences persist in a representative large-scale study. Nevertheless, the findings of this pilot study have to be interpreted as rather hypothesis generating.

More importantly, the results deliver information regarding the feasibility of a large-scale patient-reported experience and outcomes study in Schleswig-Holstein, Germany. We showed that the collection of patient-reported experience and outcome data for patient-centred quality evaluation in oncological care is feasible within the German health care system using the existing data collection structures for cancer registration purposes in Schleswig-Holstein mirrored by high participation rates of hospital units. Moreover, relatively high response rates and an acceptable average of 7% missing values per question indicate a satisfactory acceptance of the extensive survey questionnaire among cancer patients. Overall, the response patterns of German and Danish patients were largely consistent. Yet, it was shown that a substantial re-design of questions covering the diagnostic phase is necessary and not all questions can be applied as they were. Characteristics that are unique to the German or Danish health systems need to be taken into account more carefully.

In future work, adaptation efforts have to be strengthened. Shortening the questionnaire might be beneficial in order to yield even higher response rates [39, 40] and thus ensuring increased generalisability of the results. The replication of results in a representative study covering all cancer sites is necessary in order to draw generalisable conclusions about the potential strengths and weaknesses of oncological care provided in Schleswig-Holstein.

## Conclusion

The pilot study yielded satisfactory results in terms of feasibility, acceptance and applicability. Participation and completeness of answers as well as the response patterns of German and Danish patients are comparable and consistent. We conclude that the translation was successful and feasibility is given. In a future large-scale study comparisons between Denmark and Schleswig-Holstein, Germany should be possible. However, questions heavily related to the Danish health care system have to be adapted to the German setting.

## Supplementary information

**Additional file 1: Figure S1.** Flowchart of the questionnaire adaptation process.

**Additional file 2: Table S1.** Patient reports and ratings of experiences during the treatment phase at hospital.

**Additional file 3: Table S2.** Patient-reported experiences at discharge from hospital and satisfaction with overall care at hospital.

## Data Availability

The datasets used and/or analysed during the current study are available from the corresponding author on reasonable request.

## Abbreviations

EORTC QLQ-C30: Quality of Life Questionnaire by the European Organisation for Research and Treatment of Cancer containing 30 core questions
GP: General Practitioner
HLQ: Health Literacy Questionnaire
PREM: Patient-reported experience measure
PROM: Patient-reported outcome measure
WAI: Work Ability Index
