# Supplementary figures and images for: The acceptance and applicability of a patient-reported experience measurement tool in oncological care: a descriptive feasibility study in northern Germany
Source: BMC Health Serv Res. 2019 Nov 1;19:786. doi: 10.1186/s12913-019-4646-4 (PMC6825358; doi:10.1186/s12913-019-4646-4)

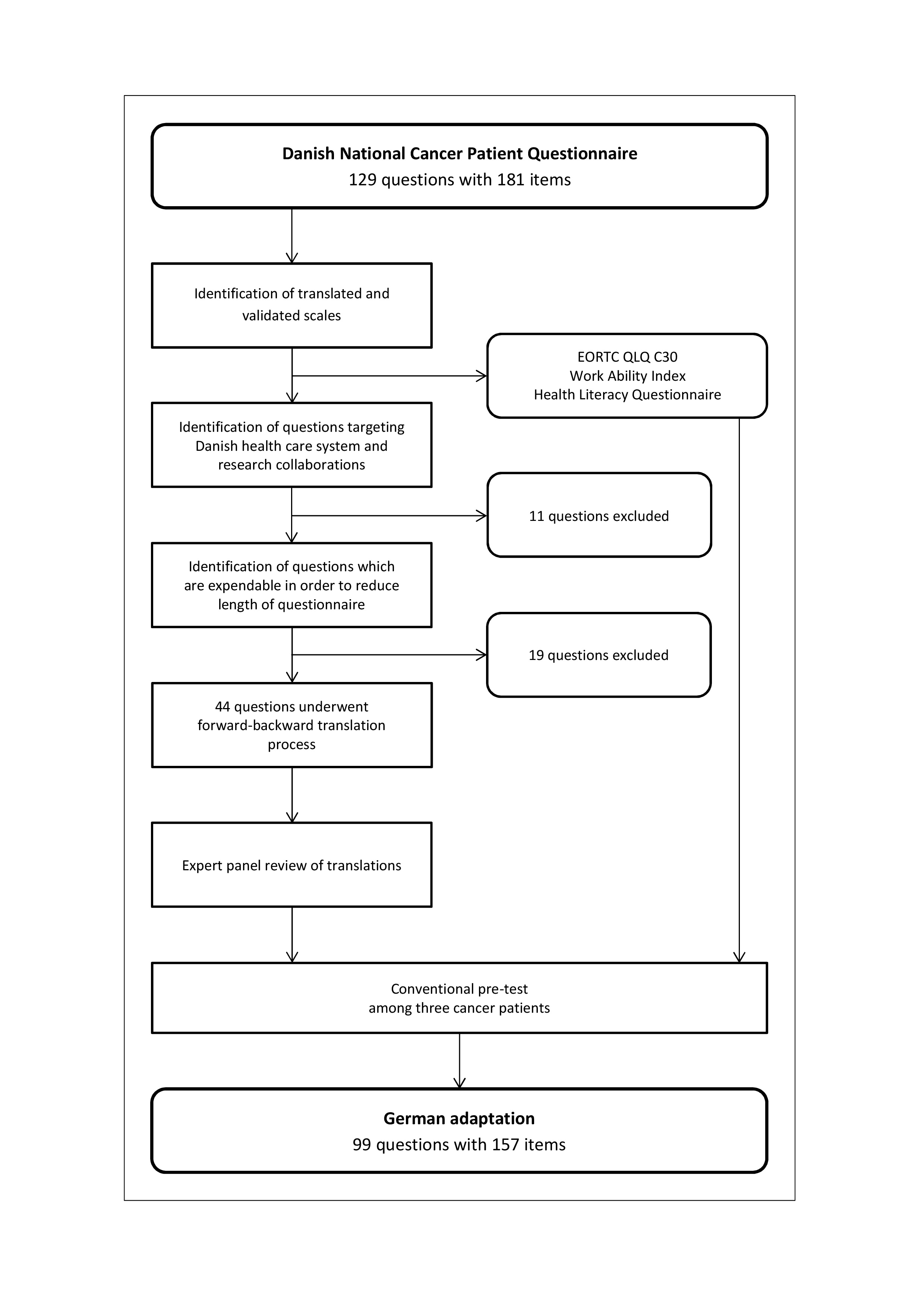

Supplement: Supplementary file 1 — Additional file 1: Figure S1. Flowchart of the questionnaire adaptation process. [file 12913_2019_4646_MOESM1_ESM.jpg]
