# Supplementary material for: The acceptance and applicability of a patient-reported experience measurement tool in oncological care: a descriptive feasibility study in northern Germany
Source: BMC Health Serv Res. 2019 Nov 1;19:786. doi: 10.1186/s12913-019-4646-4 (PMC6825358; doi:10.1186/s12913-019-4646-4)
Supplement: Supplementary file 2 — Additional file 2: Table S1. Patient reports and ratings of experiences during the treatment phase at hospital. [file 12913_2019_4646_MOESM2_ESM.docx]

**Table S1:** Patient reports and ratings of experiences during the treatment phase at hospital.

| Continuity of care | SH | DK |
| --- | --- | --- |
|  | **%** | **%** |
| 11. Which of the following statements best describes your experience at the hospital? | n=146 | n=1 783 |
| - One particular doctor was responsible for my overall treatment. | 43.2 | 31.8 |
| - The responsibility for my treatment changed between different doctors, and I have always been informed about which doctor was responsible. | 35.6 | 35.9 |
| - The responsibility for my treatment changed between different doctors, but I have not always been informed about which doctor was responsible. | 17.1 | 22.4 |
| - I have not experienced that any doctor has been responsible for my treatment. | 4.1 | 9.9 |
| 12. Did you feel that there was a clear plan for your overall treatment pathway? | n=155 | n=1 866 |
| - Yes, to a great extent | 79.4 | 83.5 |
| - Yes, to some extent | 16.1 | 13.1 |
| - To a lesser extent | 2.6 | 2.4 |
| - No, not at all | 1.9 | 1.0 |
| 13. How do you rate the number of doctors you were in contact with during treatment at hospital? | n=155 | n=1 864 |
| - Adequate | 83.2 | 69.4 |
| - Slightly too many | 9.7 | 19.3 |
| - Too many | 2.6 | 9.9 |
| - Too few | 3.9 | 0.8 |
| - Not relevant | 0.6 | 0.6 |
| 14. Have you had at least one doctor at the hospital you could reach out to if you needed it? | n=156 | n=1 821 |
| - Yes, to a great extent | 51.9 | 44.4 |
| - Yes, to some extent | 40.4 | 19.8 |
| - To a lesser extent | 3.2 | 9.1 |
| - No, not at all | 2.6 | 11.2 |
| - Don’t know | 1.9 | 15.5 |
