# Supplementary material for: The acceptance and applicability of a patient-reported experience measurement tool in oncological care: a descriptive feasibility study in northern Germany
Source: BMC Health Serv Res. 2019 Nov 1;19:786. doi: 10.1186/s12913-019-4646-4 (PMC6825358; doi:10.1186/s12913-019-4646-4)
Supplement: Supplementary file 3 — Additional file 3: Table S2. Patient-reported experiences at discharge from hospital and satisfaction with overall care at hospital. [file 12913_2019_4646_MOESM3_ESM.docx]

**Table S2:** Patient-reported experiences at discharge from hospital and satisfaction with overall care at hospital.

| Discharge and overall treatment | SH | DK |
| --- | --- | --- |
|  | **%** | **%** |
| 16. Did you feel comfortable being discharged from hospital? | n=108 | n=858 |
| - Yes, to a great extent | 61.1 | 63.2 |
| - Yes, to some extent | 36.1 | 27.6 |
| - To a lesser extent | 1.9 | 6.6 |
| - No, not at all | 0.9 | 1.4 |
| - Not relevant | 0.0 | 1.4 |
| 17. Did you get information about which symptoms you need to respond to? | n=107 | n=930 |
| - Yes, to a great extent | 34.6 | 50.4 |
| - Yes, to some extent | 36.4 | 22.5 |
| - To a lesser extent | 12.1 | 12.0 |
| - No, not at all | 9.3 | 10.3 |
| - Not relevant | 7.5 | 4.7 |
| 18. Do you know who you can contact at the hospital if you need to? | n=108 | n=963 |
| - Yes | 83.3 | 76.4 |
| - No | 16.7 | 23.6 |
| 19. Overall, how do you rate your overall care and treatment at hospital? | n=156 | n=1 843 |
| - Particularly good | 44.9 | 62.9 |
| - Good in the main | 51.3 | 34.4 |
| - Poor in the main | 3.8 | 1.6 |
| - Particularly poor | 0.0 | 0.3 |
| - Not relevant | 0.0 | 0.9 |
